# Supplementary figures and images for: The evolving role of preprints in the dissemination of COVID-19 research and their impact on the science communication landscape
Source: PLoS Biol. 2021 Apr 2;19(4):e3000959. doi: 10.1371/journal.pbio.3000959 (PMC8046348; doi:10.1371/journal.pbio.3000959)

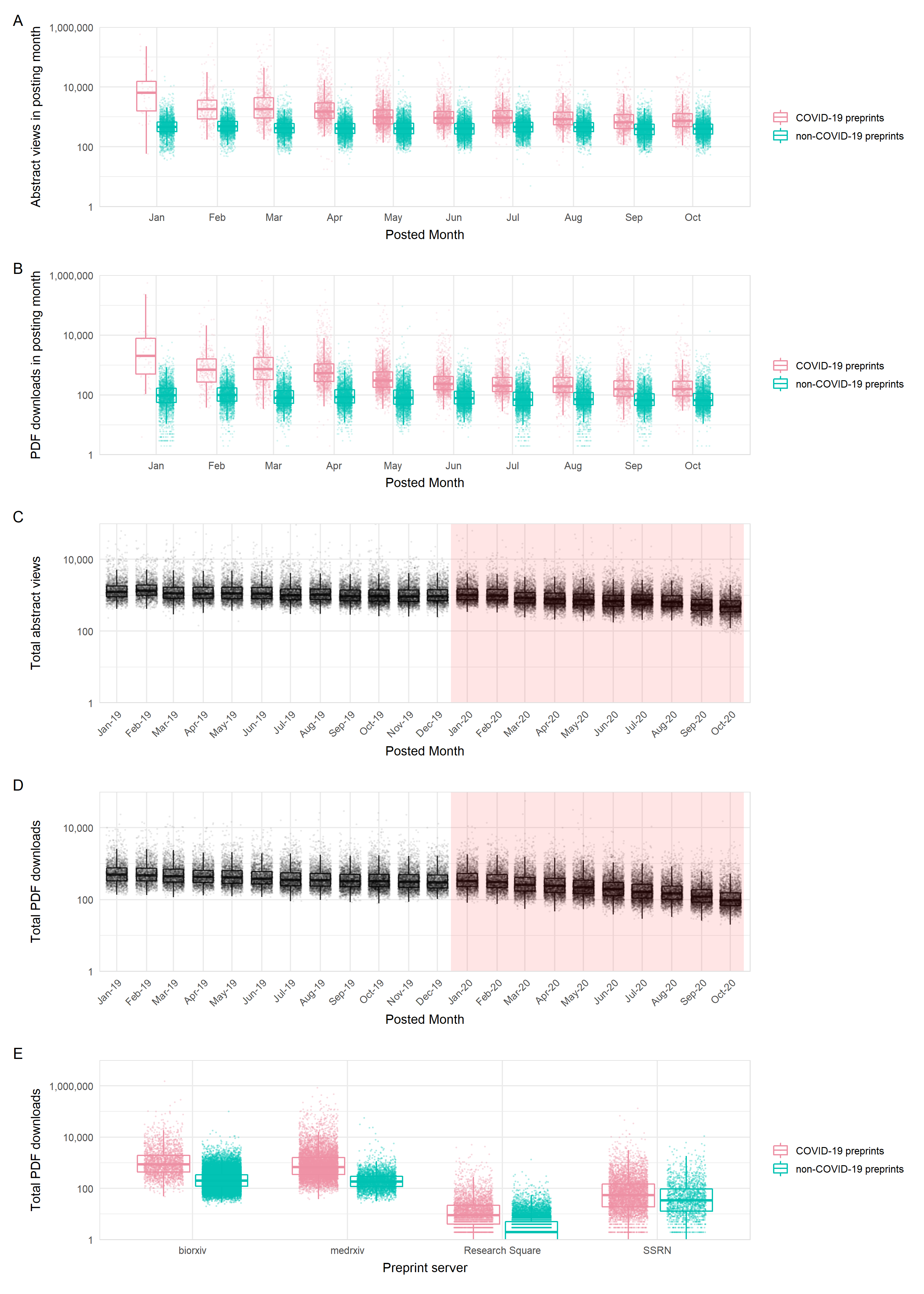

Supplement: S3 Fig — (A) Boxplots of abstracts views received by COVID-19 and non-COVID-19 preprints in the same calendar month in which they were posted, binned by preprint posting month. (B) Boxplots of PDF downloads received by COVID-19 and non-COVID-19 preprints in the same calendar month in which they were posted, binned by preprint posting month. (C) Boxplots of total abstract views for non-COVID preprints between January 2019 and October 2020, binned by preprint posting month (D) Boxplots of total PDF downloads for for non-COVID preprints between January 2019 and October 2020, binned by preprint posting month. (E) Comparison of PDF downloads for COVID-19 and non-COVID-19 preprints across multiple preprint servers. Red shaded areas in (C) and (D) represent our analysis time period, concurrent with the COVID-19 pandemic. Boxplot horizontal lines denote lower quartile, median, upper quartile, with whiskers extending to 1.5*IQR. All boxplots additionally show raw data values for individual preprints with added horizontal jitter for visibility. The data underlying this figure may be found in https://github.com/preprinting-a-pandemic/pandemic_preprints and https://zenodo.org/record/4587214#.YEN22Hmnx9A. COVID-19, Coronavirus Disease 2019. (TIFF) [file pbio.3000959.s003.tiff]

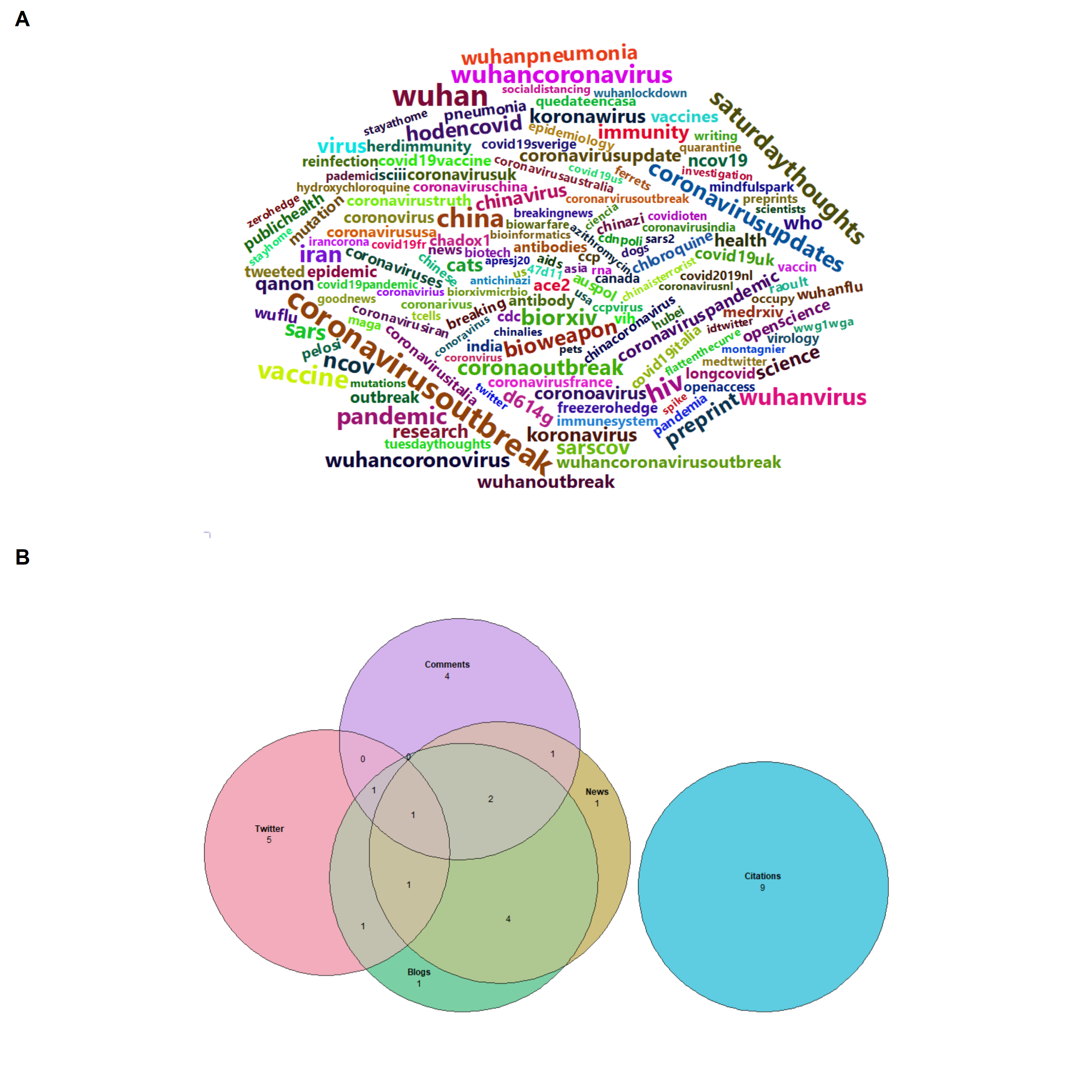

Supplement: S4 Fig — (A) Wordcloud of hashtags for the 100 most tweeted COVID-19 preprints. The size of the word reflects the hashtag frequency (larger = more frequent). Only hashtags used in at least 5 original tweets (excluding retweets) were included. Some common terms relating directly to COVID-19 were removed for visualisation (“covid19,” “coronavirus,” “ncov2019,” “covid,” “covid2019,” “sarscov2,” “2019ncov,” “hcov19,” “19,” “novelcoronavirus,” “corona,” “coronaovirus,” “coronarovirus,” and “coronarvirus”). (B) Euler diagram showing overlap between the 10 most tweeted COVID-19 preprints, the 10 most covered COVID-19 preprints in the news, the 10 most blogged about preprints, the 10 most commented-upon preprints, and the 10 most cited COVID-19 preprints. The data underlying this figure may be found in https://github.com/preprinting-a-pandemic/pandemic_preprints and https://zenodo.org/record/4587214#.YEN22Hmnx9A. COVID-19, Coronavirus Disease 2019. (TIFF) [file pbio.3000959.s004.tiff]
