# Supplementary material for: The evolving role of preprints in the dissemination of COVID-19 research and their impact on the science communication landscape
Source: PLoS Biol. 2021 Apr 2;19(4):e3000959. doi: 10.1371/journal.pbio.3000959 (PMC8046348; doi:10.1371/journal.pbio.3000959)
Supplement: Attachment — Submitted filename: Response to reviewers.docx [file pbio.3000959.s010.docx]

Dear Editor,

Thank you for the opportunity to revise our manuscript. Please find below point-by-point responses to each of the comments made by reviews. A major change we have made in the revised manuscript is to extend our dataset of preprints to cover the period January-October 2020 (the previous version covered January-April 2020). This extended period allows us to document how the preprint response has evolved as the COVID-19 pandemic has progressed. We have thoroughly re-written our manuscript to account for this new, updated, analysis. Our new analysis demonstrates lasting trends in terms of preprint access, usage and sharing over the COVID-19 pandemic.

Several of the reviewers made notes about wrong/inconsistent numbers and calculations used throughout the manuscript. This was unfortunately the result of a versioning error during submission, in which a previous version of the dataset was used. We have carefully checked all figures, calculations and statistics in the new version and are confident that the latest version is correct.

Kind regards

## Reviewer #1:

In "Preprinting the COVID-19 pandemic," Fraser et al. present a useful and timely analysis on the fate of preprints during the COVID-19 pandemic. The paper reports that preprints investigating COVID-19 are published at a higher rate than preprints on other topics, with much shorter delay between preprint and publication. The study also shows that COVID-19 preprints are much shorter and highlight interesting differences between papers at bioRxiv and medRxiv, particularly regarding screening time. This work represents an important contribution to our understanding of the role of preprints in the current pandemic and what we might expect from a future crisis. The data collection approach is logical, organized and thorough. However, there are several major issues that I recommend addressing.

MAJOR POINTS

1. The manuscript's primary dataset is preprints submitted to bioRxiv and medRxiv between 1 Jan 2020 and 30 Apr 2020. The work asks important questions, but, entering the 11th month of a pandemic in which conditions continue to change quickly, the answers presented in the paper may be representative of the pandemic, as trends may have shifted since April. The manuscript would greatly benefit from analyzing preprints from January through at least the summer, when COVID-19 preprint submissions began to decline again: [https://github.com/nicholasmfraser/covid19_preprints](https://eur01.safelinks.protection.outlook.com/?url=https%3A%2F%2Fgithub.com%2Fnicholasmfraser%2Fcovid19_preprints&data=04%7C01%7C%7C02799805ed204eab3d5608d88a2cf9f9%7C569df091b01340e386eebd9cb9e25814%7C0%7C1%7C637411273089696285%7CUnknown%7CTWFpbGZsb3d8eyJWIjoiMC4wLjAwMDAiLCJQIjoiV2luMzIiLCJBTiI6Ik1haWwiLCJXVCI6Mn0%3D%7C2000&sdata=iXCMA%2BrgjVk2%2FAEthu6bbatiECcOPCEFB7YIyaajcco%3D&reserved=0)

We have updated our analysis to include preprints posted from the beginning of January 2020, until the end of October 2020, extending the length of our analysis period from 4 to 10 months. Additionally, as the reviewer states, this enables us to capture both the peak of COVID-19 submissions, which appeared to occur in bioRxiv and medRxiv in May/June, and the period on either side of this. As such, we have revised all figures, results and discussion accordingly, as discussed in the responses to the following comments.

2. The results regarding publication rate (and publication delay) would be much more interpretable if the deadline for publication were extended significantly past the deadline for posting the preprint. Currently, the 30 Apr cutoff for preprints appears to be identical to the cutoff for publication—i.e. preprints posted on 27 Apr still count toward the tally of total preprints, but would only count toward the published preprints if they were published within 3 days of appearing online. This has at least two direct effects on the results: First, it artificially deflates the COVID-19 publication rate, particularly relative to the non-COVID-19 publication rate, which is based on a corpus that is growing at a slower (relative) pace. Second, and more importantly, it skews the "time to publication" measurement in favor of preprints that are published quickly: The paper reports that many COVID-19 preprints are published in less than 30 days, but, given the growth pattern of

COVID-19 preprints, most papers in their dataset could only have been published in less than 30 days. If the same preprints were evaluated, but the publication cutoff was extended for another 4 months, would the distribution even out? One way to examine this would be to re-evaluate publication-related outcomes after excluding preprints posted close to the publication cutoff. For example, if publication data is analyzed as of 30 Sep, it would be helpful if publication results only considered preprints posted before, say, 1 Aug, even if the other analyses consider preprints posted all the way through September.

We believe that our updated analysis negates many of these issues as we are including a much larger timeframe. We do not believe that adding a cutoff would be beneficial as we would miss those preprints published rapidly following posting to a preprint server. Even with our extended dataset, it is clear that COVID-19 preprints are being published quicker than non-COVID-19 preprints, a finding that has been supported elsewhere [1].

3. Several calculations described in the paper appear to be incorrect. While these would almost certainly be caught during re-analysis of an expanded dataset, I wanted to highlight them here as well. I couldn't find code for the analyses, so I made my best guess for how to replicate the tests given the data in the paper's associated GitHub repository.

The reviewer is correct. For each of the below cases, it appears that there was a mistake in the submission process and some older (incorrect) figures were included with the submission, except where otherwise noted. This also affected figure 2J (the reviewer also noted incorrect data below), and supplemental figures 2E and 2F. We have corrected these in the new version, apologies. Additionally, the github repo associated with this analysis (<https://github.com/preprinting-a-pandemic/pandemic_preprints>) now contains all code associated with the statistical analyses.

Line 144: The paper states, "single-author preprints were almost three times more common among COVID-19 than non-COVID-19 preprints." By my count, 205 out of 2527 COVID preprints (8.1%) listed only one author, and 288 out of 12285 non-COVID preprints (2.3%). Dividing the COVID proportion by the non-COVID proportion is 3.46, not "almost three."

We have corrected this with the new dataset. The new version reads “However, single-author preprints were ~2.6 times more common for COVID-19 (6.1% of preprints) than non-COVID-19 preprints (2.3% of preprints) (Fig. 3A)”.

Line 191: Using the data in "preprints_full_20190901_20200430.csv," I can't reproduce the reported medians here. When comparing COVID and non-COVID preprints, the text states it's 3432 vs 6143, but I get 3472 vs 6174.

We have corrected this with the new dataset. The new version reads “We found that COVID-19 preprints are on average 32% shorter in length than non-COVID-19 preprints (median, 3965 [IQR 2433] vs 5427 [IQR 2790]; Mann-Whitney, p < 0.001).”

Lines 202-204: The text says that 4% of COVID preprints were published, compared to 3% of non-COVID preprints. However, Figure 2I indicates the COVID publication rate is somewhere around 12 percent, with the non-COVID rate around 6 or 7. The provided data supports the version in the text, so it would be helpful to fix (or explain) the difference here.

The text and figures have been checked for correctness and consistency. In total we now find that 21.1% of COVID-19 preprints were published in our analysis period, compared to 15.4% of non-COVID-preprints, however the new version also explores time-dependent changes in publication rates (see Figure 4A).

Line 210: The paper reports a mean publication delay of 22.5 days for COVID preprints and 48.7 days for non-COVID preprints, but from their data, I get means of 21.0 and 32.7, a much smaller gap. The difference in means is tested using a two-way ANOVA test, but only one interaction term (COVID vs non-COVID) is clear from the description. If "source" (bioRxiv vs. medRxiv) is used as the second term, an ANOVA for "delay_in_days ~ covid_preprint+source" returns non-significant F-values for both source and covid_preprint, suggesting COVID status may not actually affect publication time—a big change from the stated results.

Here, we applied preprocessing steps to filter data to the top publishers only, and removed any erroneous negative values in the delay_in_days variable, Additionally, this analysis described an ANOVA between preprint type and publisher, i.e. delay_in_days ~ covid_preprint * publisher. We have clarified this preprocessing and model interaction within the Methods and Results, respectively:

“When calculating delay from preprint posting to publication dates, erroneous negative values (i.e., preprints posted after published versions) were ignored”

“This acceleration additionally varied between publishers (two-way ANOVA, interaction term preprint type*publisher; F9,5273 = 6.58, p < 0.001)”

Line 214: The paper describes an ANOVA using publishers, but the degrees of freedom listed (283) suggest they actually used journals, not publishers. It could well just be a typo, but there are 284 journals and only 62 publishers in the dataset. Clarification would be helpful.

The second degrees of freedom here is the df of the residuals, i.e. n – k. This is small because we subset to only the given publishers as above and is unrelated to the number of journals. This section of the results has been condensed to clarify the analyses being described.

Line 270: The text states that 6.7% of non-COVID preprints were mentioned in a news article. Of the 12,285 non-COVID preprints in the analysis period, I only see 83 with at least one mention in a news article, which is 0.68%.

We have corrected this with the new dataset. The new version reads: “In terms of coverage, 28.7% of COVID-19 preprints were featured in at least a single news article, compared to 1.0% of non-COVID-19 preprints (Fig. 6C)”

Figure 2: It appears panel 2J has incorrect data in it: It caught my eye because there are papers in the "140 days" bucket, even though the analysis period is shorter than 140 days. Reproducing the panel using the data in the repository shows a different distribution that doesn't go as far to the right.

We have corrected this with the new dataset (Figure 4C). The maximum delay between preprint posting and publishing was 298 days, consistent with the length of our analysis period (304 days).

Figure S2: I'm unable to reproduce panels E and F from this figure using the data provided. As submitted, the panel says Science has published 15 COVID preprints, for example, but I can only find 4. It says the Journal of Infectious Diseases has published 10, but I can only find 4, and so on. This may be caused by the same issue present in Figure 2J. (Incidentally, the bars in panel E are organized alphabetically, which doesn't seem like the order that would be most relevant to readers. Ordering them by value may present the information in a way that's more easily interpretable.)

We have corrected this with the new dataset (Figure 4B). The new figure is ordered by value.

4. I defer to the editor on whether this is a major issue, but I wanted to highlight several ways in which the current submission doesn't meet my understanding of the PLOS Biology requirements on data availability. First, the referenced GitHub repository provides thorough access to most of the data, but GitHub isn't intended for archival use and isn't included on the journals list of recommended repositories:

[https://journals.plos.org/plosbiology/s/recommended-repositories](https://eur01.safelinks.protection.outlook.com/?url=https%3A%2F%2Fjournals.plos.org%2Fplosbiology%2Fs%2Frecommended-repositories&data=04%7C01%7C%7C02799805ed204eab3d5608d88a2cf9f9%7C569df091b01340e386eebd9cb9e25814%7C0%7C0%7C637411273089696285%7CUnknown%7CTWFpbGZsb3d8eyJWIjoiMC4wLjAwMDAiLCJQIjoiV2luMzIiLCJBTiI6Ik1haWwiLCJXVCI6Mn0%3D%7C2000&sdata=VpVc0v%2BcabRXXCZvVdEB1DOvhnJa2J94YN5kcwcUZOo%3D&reserved=0)

The paper would benefit from depositing the code somewhere that it could be easily cited and reliably preserved. Personally, I've had a great experience with Zenodo, which has a feature that enables a direct transfer between GitHub and a free Zenodo repo:

[https://guides.github.com/activities/citable-code/](https://eur01.safelinks.protection.outlook.com/?url=https%3A%2F%2Fguides.github.com%2Factivities%2Fcitable-code%2F&data=04%7C01%7C%7C02799805ed204eab3d5608d88a2cf9f9%7C569df091b01340e386eebd9cb9e25814%7C0%7C1%7C637411273089706278%7CUnknown%7CTWFpbGZsb3d8eyJWIjoiMC4wLjAwMDAiLCJQIjoiV2luMzIiLCJBTiI6Ik1haWwiLCJXVCI6Mn0%3D%7C2000&sdata=l8Tgc0cA5a2YLU7bMCS8YgAnElPutag91juWhzFWBHw%3D&reserved=0)

Figure S3: I believe the data used for panel C is missing from the dataset. The PLOS guidelines state that "all numerical values used to generate graphs must be provided": [https://journals.plos.org/plosbiology/s/submission-guidelines](https://eur01.safelinks.protection.outlook.com/?url=https%3A%2F%2Fjournals.plos.org%2Fplosbiology%2Fs%2Fsubmission-guidelines&data=04%7C01%7C%7C02799805ed204eab3d5608d88a2cf9f9%7C569df091b01340e386eebd9cb9e25814%7C0%7C0%7C637411273089706278%7CUnknown%7CTWFpbGZsb3d8eyJWIjoiMC4wLjAwMDAiLCJQIjoiV2luMzIiLCJBTiI6Ik1haWwiLCJXVCI6Mn0%3D%7C2000&sdata=3nwLt6MIikr0M%2B%2B%2Fi11BfeCv1c6pcFqD5JxFU8y4JdI%3D&reserved=0)

Similarly, there is no data available to reproduce the results described on lines 233-243. While it looks like the data was not licensed for public release, is it possible anonymized data (e.g. lists of downloads, without any metadata attached) would be allowed, since that's effectively what appears in the figure?

Lines 215-217: The statement that "non-COVID-19 preprints had a 10.6% higher acceptance rate than COVID-19 manuscripts" is poorly supported here. While it's not hard to believe this is an accurate characterization of the data that was provided to the authors, there is no information more specific than "several publishers" about what journals this refers to. In addition, readers are not provided with any data to support the findings, nor, as required in the PLOS guidelines, are they given "All necessary contact information others would need to apply to gain access to the data."

[https://journals.plos.org/plosbiology/s/data-availability](https://eur01.safelinks.protection.outlook.com/?url=https%3A%2F%2Fjournals.plos.org%2Fplosbiology%2Fs%2Fdata-availability&data=04%7C01%7C%7C02799805ed204eab3d5608d88a2cf9f9%7C569df091b01340e386eebd9cb9e25814%7C0%7C0%7C637411273089716275%7CUnknown%7CTWFpbGZsb3d8eyJWIjoiMC4wLjAwMDAiLCJQIjoiV2luMzIiLCJBTiI6Ik1haWwiLCJXVCI6Mn0%3D%7C2000&sdata=8ksoYYUN6jFlUj7KJbV3oyRdS89Wc771arDm%2Fzs37MY%3D&reserved=0)

We have now deposited our code and underlying data to Zenodo (we’d like to express thanks to the reviewer for the recommendation). Moreover, following discussions with the preprint servers, we have now also provided the underlying data for Fig S3, although we have had to remove data from one publisher who did not return this to us in time (preprints.org). Due to continued difficulties in obtaining the data from publishers, we have chosen to remove the statement and data referred to in lines 215-217.

MINOR POINTS

Line 86: The paper states that COVID-19 preprints "are reviewed faster than their non-COVID-19 counterparts," but that isn't the only explanation for the observed differences. Given the stakes, it's not impossible that preprint authors were just less likely to post a preprint until they knew they had a done deal at a journal. For authors scared (rightly or not) of getting scooped, posting a preprint 24 hours before your peer-reviewed article goes live may be a way to drum up attention and appear "open" without any risk. Unless there is a way to demonstrate that authors deposit COVID and non-COVID preprints at the same point in the publication process, the statement that they are "reviewed faster" seems to make a large interpretive leap when a phrase like "spend less time on bioRxiv prior to publication" is better justified. This may be a moot point if changes are made regarding Major Issue 2 above.

We softened the text here to instead read “COVID-19 preprints […] are published in journals with a shorter delay following posting than their non-COVID-19 counterparts”. Whilst it may be the case that some authors choose to post a preprint very shortly before an article appears in a journal, bioRxiv and medRxiv do not allow preprints to be posted after acceptance (so it is unlikely that a preprint would appear ~24 hours before going live on a journal page), and the distributions shown in Figure 4C would indicate that this is not generally the case (otherwise we would expect a spike in the 0-10 day bin). A separate analysis by Horbach [2], which is mentioned in our discussion section, also looked at publishing timelines of COVID-19 research (not limited to preprints) using submission, acceptance and publication dates listed on journal websites, and concluded that the time between journal submission and publication for COVID-19 research was reduced by 49%, predominantly driven by faster peer-review times.

Line 98: In multiple places (lines 31 and 109, Figure 1B), the text references the number of published papers related to COVID-19, but it's unclear where this information comes from. The legend for figure 1 says "Journal data in (B) is based upon data extracted from Dimensions," but the paper would benefit from elaboration in the Methods section regarding the search strategy and when the search was performed.

We have added a section to the methods entitled “Journal Article Data” elaborating on this.

Lines 185-187: The paper states that "COVID-19 preprints did not discernibly differ in number of versions compared with non-COVID-19 preprints," using as evidence that both categories have a median of 1. However, Figure 2C shows a noticeable difference in the distributions. Testing the difference between groups using something like the Mann-Whitney test would enable including a definitive statement.

A supporting test and further description have now been added as suggested.

Line 192: The paper states that the difference in preprint length between COVID and non-COVID papers "supports the anecdotal observations that preprints are being used to share more works-in-progress rather than complete stories." However, given the accelerated publication rate of COVID preprints, it seems likely that this could also just indicate that for COVID, the bar for a "complete story" is lower. This isn't a necessary analysis, but if the authors are interested, this section would be improved by an analysis of preprint length among PUBLISHED preprints: Do shorter preprints have a longer delay before publication? If so, I think that would be much more supportive of the idea that people are sharing results as they work. However, if short COVID-19 preprints are published just as quickly as longer ones, that suggests a different story.

We have expanded the analysis with a multivariate mixed-effects model of word counts (also based on comments from Reviewers 2 and 4, below) to consider additional variables that may be associated with longer/shorter preprints (Supplemental Model, Supplemental Table 2 & 3). We find that a difference in preprint length persists between COVID-19 and non-COVID-19 preprints, when adjusted for number of authors, published status and publishing delays, date of posting, country of corresponding author and bioRxiv subject categorisation, and that the average adjusted absolute difference in word count is at least as large as that reported in the simple univariate test in the main results. Specifically regarding publishing delays, we find that shorter preprints have a *shorter* delay before publication; this effect was present for both COVID-19 and non-COVID-19 preprints, but was most pronounced for COVID-19 preprints (an additional 100 words was associated with increased average delays of 16.5 days vs 64.9 days respectively). We believe this indicates that preprints are being posted as short reports, rather than works in progress. We have removed this statement from the manuscript.

Line 195: The text states that the difference in total references between COVID and non-COVID papers reflects "the new, emerging COVID-19 field and dearth of prior literature to reference." However, particularly given the dramatic length difference, maybe shorter, more straightforward COVID preprints simply require less supporting references—if an average non-COVID preprint reports the results of, say, 3 major experiments, it would probably require more background and support than a COVID preprint only reports one experiment. The most straightforward fix for this is to remove some of this over-interpretation, but it may be testable by evaluating something like "references per word."

We have now tested for differences in references:word count ratios (see Rebuttal Figure 1) and indeed find that there as many references in COVID-19 preprints as expected given their shorter length. The Results have been updated to note this: “COVID-19 preprints also contain fewer references than non-COVID-19 preprints, though not fewer than expected from preprint length, as little difference was detected in reference:word count ratios (median, 1:103 vs 1:101; p = 0.052)”

**Rebuttal Figure 1.** Density plot of reference:word count ratios for COVID-19 and non-COVID-19 bioRxiv preprints. X axis depicts log-scale of increasing reference:word counts.

Line 486: More documentation would be helpful regarding how the preprint publication dates were determined. The text specifies that they were retrieved from Crossref, but the Crossref API provides several different "publication" dates that do not always match. Since there is not an explicit connection between publication date and the date Crossref receives the data, it would also be helpful if the paper specified the dates that the publication data was pulled.

We used the Crossref “created” field as the date of publication - this refers to the date on which the DOI and metadata record in Crossref were originally created. Whilst there are several publication dates in Crossref that we could choose from (e.g. the “published-online” and “published-print” fields), the “created” field has previously been suggested as a good proxy for the first online availability of an article [3,4] and has the advantage that it always contains a full date in YYYY-MM-DD format, as opposed to the “published-online” or “published-print” dates, which sometimes only contain month or year information. This is now documented in the new version of the manuscript (“With respect to publication dates, we use the Crossref “created” field which represent the date on which metadata was first deposited and has been suggested as a good proxy of the first online availability of an article [53,54]”). Dates on which metadata was pulled is documented in the methods (“Preprint metadata, and metadata related to their linked published articles, were collected in the first week of December 2020.”).

OTHER SUGGESTIONS

The notes below are only intended as friendly suggestions and are not critical to the integrity of the paper.

Lines 27-37: I found it striking that the abstract does not actually describe any results. Readers may be more likely to read on if they're given a better idea of what kind of analysis is to come.

We have updated our abstract and included an overview of the results.

Line 109: The word "published" here is ambiguous: It sounds like the 6,753 preprints included in this total were preprints that were all subsequently published in journals, but, if I'm reading it correctly, this conflates posting to a preprint server with "publishing." Given that the paper deals with preprints that later appeared in journals, it would be beneficial to rephrase this.

We updated this section of the introduction to reduce ambiguity between terms such as “publishing” and “articles” when discussing journal articles and preprints.

Line 155: The phrase "as an expectation" tripped me up here—perhaps "as expected" would be more clear?

Removed “as expected” from the manuscript.

337: This sentence suggests the use of preprint servers has been "encouraged by funding bodies requiring COVID-19 research to be open access," but the call to make research open access seems almost separate from the push for people to post preprints—that is, it's not clear to me that posting a preprint prior to publication would satisfy Wellcome's commitment that "all peer-reviewed research publications relevant to the outbreak are made immediately open access." Given that both cited examples strongly encourage preprints but only Wellcome's mentions open-access publication, it may be better to edit this sentence to remove the phrase "requiring COVID-19 research to be open access."

We have removed this from the manuscript

Lines 402-404: Seems like this manuscript is citing its own preprint. This is the first I've seen this, and I'm not sure what is the rationale. If there is an analysis that was included in the preprint version but not the current version, yet still important enough to cite, it might make sense to include the analysis in the current manuscript.

The data referred has been expanded and is currently being written as an independent study which will be shared soon. The reference has been removed.

Lines 529-531: The citations of preprints seems very relevant and is important enough to move up to the results section. In addition, the phrase "all preprints" is unclear here, since the manuscript includes references to multiple preprint servers. It appears this refers to the bioRxiv and medRxiv preprints posted in September 2019 or later; it would be helpful to clarify that.

We have altered the wording to clarify what preprint servers we refer to. Lines 542-545, now read:

“For investigating the rates at which preprints are used, shared and cited, we collected detailed usage, altmetrics and citation data for all bioRxiv and medRxiv preprints posted between 1st September 2019 to 30th April 2020 (i.e. for every preprint where we collected detailed metadata, as described in the previous section).”

Line 573: It's not clear what method was used for multiple test correction here. The impression that I get from the multcomp documentation is that the glht.summary() function has multiple sophisticated options. It's possible I'm misreading this, but more clarity would be appreciated.

As described in the text, we use Tukey’s honest significant difference, which is available as an option for multiple comparison methods in the glht() function. We have added further detail and note that we now supply all code associated with the statistical analysis.

Figure 1: The y-axis label in panel B looks at first glance like it may refer to a ratio, though I think it actually means "articles OR preprints." Would "Manuscripts" be more clear as a label?

We replaced the “/” in the y-axis label with “&” (Figures 1A and 1B)

Figure 2: Panel B has been effectively scrambled by the lopsided number of preprints processed between medRxiv and bioRxiv, data that is much better visualized in Figure S2 (panel B). Right now, Figure 2B makes it look as if COVID preprints took far longer than usual to be screened, while the results (and Figure S2b) show that there wasn't a big COVID effect, and it's just that COVID preprints were more likely to show up on medRxiv, where screening always takes longer. I suggest replacing Figure 2B with Figure S2B, which is a little more cluttered but far more interpretable.

For each of the panels in Figure 2 (except word/reference counts that only apply to bioRxiv), we show the same data split by individual preprint servers in Supplemental Figure 2 (to maintain consistency and reduce clutter of too many panels). However, to avoid confusion we have added sections to the text describing in more detail the differences between the two servers: “A minor but detectable difference was observed between screening time for COVID-19 and non-COVID-19 preprints (Fig. 2B), though these differences appeared to vary with server (two-way ANOVA, interaction term; F1,83333 = 19.22, p < 0.001). Specifically, screening was marginally slower for COVID-19 preprints than for non-COVID-19 preprints deposited to medRxiv (mean difference = 0.16 days; Tukey HSD, p < 0.001), but not to bioRxiv (p = 0.981). Larger differences were observed between servers (Supplemental Fig. 2B), with bioRxiv screening preprints on approximately 2 days quicker than medRxiv for COVID-19 and non-COVID-19 preprints (both p < 0.001).”

Figure 2D contains so much interesting data, but is almost impossible to read because of the delineation between first-time authors and returning ones. This panel may be better as a supplementary figure. At the author's discretion, I'd suggest making a scatter plot similar to the one in Figure S2C, plotting COVID vs. non-COVID percentages, leaving the distinction of first-time authors to a supplement.

We have now re-created this figure and hope that the data is clearer.

Panel 2I seems to be an unnecessary use of space—I think readers can conceptually compare one number against another, slightly smaller number without a picture.

We removed this simplistic panel in the new version, and added a new panel showing differences in publication rates as a function of time (Figure 4A).

## Reviewer #2:

[identifies himself as Euan Adie]

Fraser et al. have produced a thorough, detailed analysis of preprints relating to COVID-19 in 2020 and compared them to non-COVID preprints in the same period and earlier.

I'm impressed by the range of the data examined and by the quality of the associated, documented code for the analysis the authors have placed on github, though I didn't re-run the analysis for myself.

We've unquestionably seen a change in the use of preprints during the pandemic and the breadth & wide scope of this study makes it novel and significant enough for publication.

That said I did find myself wanting a clearer picture of what some of the data means, particularly around whether or not how many COVID-19 preprints are genuine work of a standard equivalent to what would normally be submitted to preprint servers vs more lightweight but still high quality articles vs opportunistic "spam" articles. The manuscript touches on some of these points but left me unclear.

My comments in order of appearance rather than importance, (6) is the only revision I'd consider essential:

1)	COMMENT Line 70: preprints aren't only -scientific- manuscripts, you mention humanities etc. preprints later on

We replaced the word “scientific” with “scholarly” here to be more inclusive.

2)	COMMENT Line 80: first time preprints have been widely used to communicate during epidemic: "widely" is doing a lot of work here… as we don't know e.g. what proportion of all researchers working on Zika used preprint servers. Maybe better to say widely used outside of specific communities?

We have altered the wording of this sentence as suggested, it now reads “The COVID-19 crisis represents the first time that preprints have been widely used outside of specific communities to communicate during an epidemic.”

3)	COMMENT Line 109 - more than 16,000 articles published, a large proportion preprints: as a number of preprints go on to become published articles it'd be good here to highlight the number that are preprints OR published versions of preprints rather than just the former.

Calculating the proportion of preprints that are published would require sophisticated matching processes that are beyond the aims of this study. To address this point, we added a line in the results section that a proportion of preprints are also published in journals, and thus some double-counting may occur.

4)	QUESTION Line 112 -SSRN - I'm not familiar with the SSRN workflow but do notice that The Lancet "First Look" papers live there [https://www.ssrn.com/index.cfm/en/the-lancet/](https://eur01.safelinks.protection.outlook.com/?url=https%3A%2F%2Fwww.ssrn.com%2Findex.cfm%2Fen%2Fthe-lancet%2F&data=04%7C01%7C%7C02799805ed204eab3d5608d88a2cf9f9%7C569df091b01340e386eebd9cb9e25814%7C0%7C0%7C637411273089716275%7CUnknown%7CTWFpbGZsb3d8eyJWIjoiMC4wLjAwMDAiLCJQIjoiV2luMzIiLCJBTiI6Ik1haWwiLCJXVCI6Mn0%3D%7C2000&sdata=4DDJ1nSkEw%2FzD8TGJad%2BfH549WIa5xiFZquDskzhzoM%3D&reserved=0) … were these treated as preprints, and did any other medical journals introduce "one click" ways to deposit papers as preprints in 2020? Basically were any new workflows introduced that might have influenced author choices?

The Lancet “First Look” papers were included in the dataset of preprints from other repositories (note that this dataset is collected externally to the study here, although the first author is involved with both). The authors are not aware of any technical changes (e.g. “one-click” depositing systems) that were introduced by medical publishers, but there were a number of changes in peer-review policies that may have supported author choices to submit to a journal - e.g. eLife adapted their policies to make preprint depositing on bioRxiv/medRxiv the default choice, and curtailed requests for additional experiments during peer review to reduce potential publication delays [5].

5)	COMMENT Line 181 - different types of license being adopted for COVID-19 work: I'd be interested in some brief discussion around why this might be. e.g. are there pharma collaborations or is it connected to how new to preprints authors are? It doesn't gel 100% with authors wanting to be as open as possible - maybe it's just speed that's important to them?

We can only speculate on this point and did not address this directly in the manuscript. Our analysis found that many authors of COVID-19 preprints are new preprint authors (rather than authors who have posted preprints in the past). It may therefore be a lack of knowledge on what each license represents and allows than a conscious decision – as was found in a previous survey by ASAPbio that we referenced (<https://github.com/asapbio/licensing>). We also now show license types split by the two preprint servers (bioRxiv vs medRxiv, Supplemental Figure 2D), and also observe little difference in the license types chosen.

6)	ESSENTIAL SUGGESTED REVISION Line 193: support anecdotal evidence that preprints are being used to share works in progress: I'd really like to see this expanded upon here or in the discussion, as it relates also to the quality question you raise around line 201 and later on too… it seems like it would make interpreting other aspects of the data easier. Specifically, does the data suggest that (a) COVID-19 preprints are mostly opportunistic short works or genuinely works in progress, with only one version, that then get submitted to journals with few changes (which may explain the lower acceptance rate, and is what's implied at first)? or (b) do they usually undergo significant changes between preprint and final published version (on line 401 it's asserted that the preprints are of relatively good quality, because the acceptance rate is only a little lower)? In the latter case authors may not engaging with versioning or perhaps publishers have lowered standards for COVID related work, which would be good to know. You start addressing the opportunistic part by looking at changing fields which I think is a good start. I realize that as you say it's very difficult to assess the "quality" of a preprint… perhaps you could get a feel for things by assessing the number of changes in the final published version, for a random subset of the articles?

The original version of our preprinted manuscript contained some analysis on “quality” of preprints compared to their published counterparts. Ultimately, we decided to expand this analysis into a separate paper which will be shared soon. We have included some of the analysis here to answer this point (Rebuttal Figure 2). In this analysis, we compared all COVID preprints posted between January and April 2020 that had also been published within this timeframe (~4% of COVID preprints and 3% of non-COVID preprints). We first analysed abstracts by calculating a change ratio (number of changes / length of abstract) (Rebuttal Figure 2. A). This demonstrated that abstracts of COVID-related articles had more textual changes than non-COVID articles. We further utilised a second method for calculating a ratio of the number of changes between abstracts using Microsoft Words compare functionality (Rebuttal Figure 2. B). To investigate the context of these changes, we manually annotated all abstract pairs scoring the type of change (No change, altered wording leading to a softening or strengthening of the main conclusions, major changes to the main conclusions). Most articles had either no change or minor changes to strengthen or soften the conclusions (approx 85% independent of COVID or non-COVID, (Rebuttal Figure 2. C)). This confirms that the majority of the textual changes observed with the change ratio are small alterations that, whilst aid in strengthening or softening the conclusions, do not alter the main conclusions or data in the abstract. We next assessed the numbers of panels and tables in the full text. There were no significant differences between matched pairs but COVID articles had fewer total panels and tables than non-COVID articles (Rebuttal Figure 2D). This compliments our data on word counts (Fig. 2E, main manuscript). In determining if panels had been added or removed (or moved) between a preprint and published article, we found that there were greater alterations in COVID articles (Rebuttal Figure 2. E). Despite this, a more detailed analysis revealed that these changes were negligible or simple re-arrangements. Where changes were more significant, this was mostly due to added content in the published version of the article (~20% of COVID articles and ~16% of non-COVID articles, (Rebuttal Figure 2. F)). Taken together, this data suggests that, independent of COVID status, preprints do not significantly differ in their top-level conclusions and figure composition from their published counterparts, data that has been supported elsewhere [6].

**Rebuttal Figure 2. Preliminary analysis of changes between preprints and published articles.** (A-C) Analysis of abstracts for preprint-paper pairs. (A) Change ratio computated by difflib analysis. (B) Change ratio calculated from Microsoft Word compare documents feature. (C) Overall score for abstracts. (D-F) Analysis of panels and tables for preprint-paper pairs. (D) Total numbers of panels and tables. (E) Difference in the number of panels and figures between preprint and paper. (F) Overall categorisation for the changes in panels and tables between preprint and paper.

7)	SUGGESTED REVISION: Line 194: COVID-19 preprints have fewer references: are you controlling for number of words? If the articles are 44% shorter it stands to reason that they should also have fewer references, it may be that the articles are more focused or just don't have scope for many citations. I don't think we can say that it reflects only the dearth of prior literature.

As per our response to reviewer 1: We have now tested for differences in references:word count ratios (see Rebuttal Figure 1) and indeed find that there as many references in COVID-19 preprints as expected given their shorter length. The Results have been updated to note this: “COVID-19 preprints also contain fewer references than non-COVID-19 preprints, though not fewer than expected from preprint length, as little difference was detected in reference:word count ratios (median, 1:103 vs 1:101; p = 0.052)”

8)	SUGGESTED REVISION: Line 208: we see faster publication times for COVID-19 preprints: again, would be interesting to see this controlled by article length. Shorter articles with clearer, short hypotheses and opinion pieces will be easier to review and to a certain extent copyedit than longer, data heavy papers.

We tested the influence of article length by constructing a simple regression with publishing delay as the outcome variable and COVID/non-COVID preprint type and word count as predictor variables. Although both were significantly associated with article length (t = -12.5, p < 0.001; t = 3.6, p < 0.001, respectively), coefficient estimates suggested a similar difference in publishing delay when adjusting for word count (40.3 days) to that reported in our results (48 days). Note that as in our response to Reviewer 1, we also use multivariate regression to investigate relationships between word count and publishing delay, though these models use word count as the outcome variable (Supplemental Model, Supplemental Table 3).

9)	QUESTION: Line 219: was there any anecdotal evidence from MedRxiv / bioRxiv about where the abstract views were coming from? For example direct links from the websites of public health bodies.

We do not have access to this data and are unaware of any evidence surrounding this, so unfortunately cannot address this point. Of course, we do see in our data the strong usage of preprints in social media and news media, so some views are likely directed from those sources.

10)	COMMENT: Line 339: I think it's too early to say if the change is permanent or related to the specific circumstances of the pandemic

We have altered this in the manuscript: “Our results show that preprints have been widely adopted for the dissemination and communication of COVID-19 research, and in turn, the pandemic has greatly impacted the preprint and science publishing landscape”

11)	SUGGESTED REVISION: Line 376: Marked change in journalistic practice: I suspect this is correct, but it's hard to say without data on *why* papers were picked up more by the news: it could also be because university press offices that were previously only worked with press officers at high impact journals have suddenly become interested in COVID-19 preprints and so scan bioRxiv / medRxiv (or perhaps medRxiv has started reaching out to journalists directly?) and that some researchers are very keen to see public engagement around their COVID work. There are few science journalists and it is rare for journalists

We are not aware of any data that directly addresses this point. There is one study confirming that there has been a change in journalistic practice in relation to COVID-19 preprints but this does not expand on why this has occurred [7]. We have noticed an increase in press releases from universities for preprints (and a number of high-profile press-releases from companies developing vaccines). This may be one reason for journalists utilizing preprints more.

(note: it appears that this comment was cut-off).

## Reviewer #3:

The manuscript by Fraser et al. is very interesting inasmuch as it documents how many manuscripts on COVID-19 have been shared during the early months of the pandemic. However, the manuscript by Fraser et al. could be easily misread at COVID-19 being special and preprints fulfilling a unique need of the scientific community. While the authors are transparent in communicating their potential conflicts of interest, the latter framing would not seem necessary for the manuscript to be interesting.

My main concerns are well encapsulated in the statement of the abstract "Although the last pandemic occurred only a decade ago, the way science operates and responds to current events has experienced a paradigm shift in the interim. The scientific community responded rapidly to the COVID-19 pandemic, releasing over 16,000 COVID-19 scientific articles within 4 months of the first confirmed case, of which 6,753 were hosted by preprint servers.". This statement and similar impactions throughout the manuscript suggest that the way science operated changed and that this was tied to preprints.

However, I do not see the manuscript of Fraser et al. as providing evidence for a change in the way science is conducted aside from acknowledging that the volume of research dedicated to COVID-19 is large, and larger than for past pandemics. On a philosophical note, the manuscript does not follow's Kuhn's definition of paradigm shift (that would be more similar to a declination). On a scientometric level, biomedical scientists have already been very responsive toward epidemics and given SARS, MERS and other global threats a disproportional share of their attention. This could be seen, for instance, by determining the citation metrics of publications (controlled for years) for publications in MEDLINE of each individual MeSH term. Such an analysis places MeSH terms corresponding to emergent pathogens as the most-cited MeSH terms of at least the last ~15 years, generally even at the peak position of all MeSH terms (if one excluded MeSH terms occurring only in a handful of manuscripts). One may interpret this as no single set of topics of biology having received as much interest of scientists as pathogens causing pandemics. An alternative reading of the manuscript of Fraser et al. could thus be that scientists did - and do - redirect their attention toward emergent pathogens and pandemics - but that the volume of research on COVID-19 is higher than for other emergent pathogenes/epidemics due to other reasons (e.g.: fraction of people in research-heavy countries that are affected?). Likely, one may conclude that preprints have not been necessary for emerging pathogens to draw more attention among scientists than any other topic.

We thank the author for their comments. We believe that our data does show that the response to COVID-19 is different to that of previous pandemics and current research practices. It is particularly important to note that preprint usage was very limited for most previous epidemics. For example, Supplemental Figure 1D demonstrates a more rapid and larger response to COVID-19 than to previous recent epidemics, particularly with regards to preprint usage. Moreover, COVID-19 preprints are experiencing significantly increased usage compared to non-COVID-19 preprints posted during the same period. We also find that many researchers are posting preprints for the first time in response to the pandemic (Fig. 3C); further suggesting a change in publishing practices.

We have found that journalistic practices are also changing to report more preprints than previously (Fig. 6); indeed, the finding that “28.7% of COVID-19 preprints were featured in at least a single news article, compared to 1.0% of non-COVID-19 preprints” demonstrates a very clear change in news reporting. Our findings that COVID-19 preprints are being used in policy documents (Fig. 6) further supports the notion of wide-ranging changes in practices across disciplines.

With regards to changes in how science is operating, other studies have demonstrated an increase in the usage of preprint servers [8,9] and there have been numerous examples of a shifting towards greater adoption of open-access principles and initiatives, such as those by eLife [10]. We believe that many of these changes have been accelerated by the pandemic and this is reflected in our data.

We believe that our data demonstrates that preprints are fulfilling a unique need of the scientific community and supports the numerous statements that have recently been made in editorials and by prominent public to that effect.

Other comments:

When comparing COVID-19 preprints against others it remains unclear from the methods section and text, whether all comparisons (e.g.: also license type, number of versions of preprints, lengths of texts) are restricted to preprints posted in the same observational period as COVID-19 preprints (which appears introduced as a statement in the context of Fig 2I). In case that the range of dates allowed for non-COVID-19 preprints differed, the interpretation surrounding many elements of Figure 2 might change.

We have now clarified this in the text in both the results: “We compared attributes of COVID-19 related preprints posted within our analysis period between 1^st^ January 2020 and 31^st^ October against non-COVID-19 related preprints posted in the same time frame”, and in the methods: “Note that this time period was chosen to encapsulate a 10-month analysis period from 1st January to 31st October 2020, in which we make comparative analysis between COVID-19 and non-COVID-19 related preprints (N = 44,503), as well as the preceding year from 1st January to 31st December 2019 (N = 30,094), to use as a pre-COVID-19 control group”

The authors show differences in reception of COVID-19 vs non-COVID-19 manuscripts (Figure 3), which they interpret as "extensive access". While they rule out some possible alternative scenarios, it remains unclear to which extent this reception is driven by scientists using preprints differently in the context of COVID-19 vs. preprint servers (or secondary sites, such as covidpreprints run by one of the authors) prioritizing the visibility of COVID-19 manuscripts over others (e.g.: bioRxiv has a bold red link to "COVID-19 SARS-CoV-2 preprints, suggesting that there are active efforts to help COVID-19 preprints to gain more visibility). Maybe there is data to make a more stringent statement, otherwise, I would recommend rephrasing.

bioRxiv did not add a banner/COVID collection until mid-March 2020 (comparing https://web.archive.org/web/20200315071916/https://www.biorxiv.org/ to https://web.archive.org/web/20200316101739/https://www.biorxiv.org/). Moreover, the covidpreprints website was not created until later in the year. Our data shows that preprint access was increased for COVID-19 preprints before March, suggesting that the presence of a COVID-19 collection or the covidpreprints website were not having a strong influence on usage of these preprints. It is however entirely possible that some traffic was prioritized to COVID-19 preprints once these “flags” were in place.

The data as shown in panels Fig 4A-E provides very little information not contained in the text since distributions are similar, sometimes have the same median, and there are many dots - but it remains unintuitive for reader if there would be a relative change (as total number of dots could be dominated by total number of preprints in each category). Possibly more information could be conveyed by making a cumulative plot (or survival analysis) with increasing values (now y) as thresholds (on x), and plotting two different lines (COVID-19 and non-COVID 19). As an additional, related challenge around these panel, it appears that likely the statistical tests given in the main text should be replaced by non-parametric test, or replaced by tests that do not test differences in the centrality (as dynamic range is small) - but instead test (e.g.: via Fisher's exact test) whether proportions of preprints with at least one y-value (e.g.: one citation) would differ between COVID-19 and non-COVID-19 preprints.

We changed these plots to provide more context – we now show (in Figures 6A-F) the proportion of COVID-19/non-COVID-19 preprints that receive at least one citation/mention in each indicator, binned by week of publication. This gives a clearer overview of how the usage of preprints has changed in each indicator over time. We kept the previous figures as an inset, to still be able to compare total counts of each indicator per group.

With regards to statistical tests, we agree that the usage distributions are heavily skewed, however nonparametric tests would be too simple to include a time dependency in. Similarly, we consider the resolution of exact counts to be a strength of our usage data, and comparisons of “zero versus any” as proposed would substantially weaken this. We believe our approach in fitting a regression with negative binomially distributed errors is sufficient; such a distribution is flexible and accommodates for extreme overdispersed count data [11].

For Figure 4G,H it is unclear whether the inferred statement on higher correlations among COVID-19 truly reflect different correlations, or a higher number of preprints with non-zero values among the COVID-19 group.

The nonparametric correlation method used makes no distributional assumptions. To investigate the effect of non-zero values, we repeated all correlations excluding zeroes in the respective variables in each case. Although the strength of correlations among non-COVID-preprints increases, comparatively higher correlations remain among COVID-19 preprints (see Rebuttal Figure 3)

|  |  |
| --- | --- |

**Rebuttal Figure 3.** Spearman's correlation matrix as in manuscript Figure 6H and 6I after exclusion of zero values in calculating each individual pairwise correlation. Indicators shown are that of manuscript Figure 6, as well as abstract views and pdf downloads for COVID-19 and non-COVID-19 preprints.

The discussion spares possible criticisms around preprints. First, the tournament-type economics of science that requires scientists to accumulate reputation may force researchers to publish on preprint servers due to the risk of being scooped rather than because of their perception of the usefulness of preprint servers, and thus contribute to a research culture that could be perceived unfavorably (e.g.: along "publish-or-perish"). Second, scientific disciplines can slow down in their rate of innovation if they grow too big (Chu et Evans, 2018, ScArXiv). In this sense a larger volume of publications (in manuscripts, but further increased by preprints) may be expected to lead to more conservative research and rather limit the overall progress of scientific fields (besides size).

The discussion section misses the possibility that preprint servers are not neutral services, but themselves act in a way that could prioritize COVID-19 (e.g.: by highlighting COVID-19 publications as bioRxiv does).

We agree that the risk of being scooped may be one reason why scientists are turning to preprint servers. However, this reason alone does not explain the large increase for COVID-19 preprints but may be one reason that preprint servers are increasingly being used [9,12]. Additionally, preprint servers highlighting COVID-19 preprints may also result in a higher traffic and usage. However, bioRxiv did not add a banner/COVID-19 collection until mid-March 2020 (comparing https://web.archive.org/web/20200315071916/https://www.biorxiv.org/ to https://web.archive.org/web/20200316101739/https://www.biorxiv.org/). Moreover, the covidpreprints website was not created until later in the year. Our data shows that preprint access was increased for COVID-19 preprints before March, suggesting that the presence of a COVID-19 collection or the covidpreprints website were not having a strong influence on usage of these preprints. It is however entirely possible that some traffic was prioritized to COVID-19 preprints once these “flags” were in place but not enough to produce the results we report.

Minor:

Extending the analysis beyond April 30th would be interesting as journals and the scientific community had more time to adopt. Based on Figure 3A, B, which shows diminishing differences between COVID-19 and non-COVID-19 preprints over time, it remains unclear whether the findings reported by the authors throughout the manuscript only refer to the first weeks of a pandemic (a time-period that would be very important, and where preprints might be particularly relevant), or a "shift" as they imply in the discussion section.

In the new version we have updated the dataset to include all preprints posted between 1^st^ January and 31^st^ October – this point was also raised by other reviewers.

"Escalating demands made by reviewers and editors are lengthening the publication process still further [8,9]." isn't backed up by the references, and might rather mirror a common perception, and topic of further study.

We have removed this sentence from the manuscript

While it would seem unlikely to change the overall findings, the comments on other RNA viruses might be incomplete as the keyword-based matching used by the authors uses fewer synonym than for COVID-19 and excludes most synonyms provided by NCBI Taxonomy, which is a reference database for the nomenclature of organisms.

We use fewer synonyms for these viruses as they were already known and well-described before their large outbreaks in question (in contrast to SARS-CoV-2, which was given many names during the early outbreak phase). Our search terms are inclusive of all synonyms for these viruses as defined by NCBI Taxonomy as follows:

Zika virus, acronym ZIKV, no equivalent names (https://www.ncbi.nlm.nih.gov/Taxonomy/Browser/wwwtax.cgi?mode=Info&id=64320)

Zaire ebolavirus, acronym ZEBOV, equivalent name ‘Zaire Ebola virus’ (https://www.ncbi.nlm.nih.gov/Taxonomy/Browser/wwwtax.cgi?mode=Info&id=186538)

The analysis of the paragraph between lines 135-141 visually appears at odds with the referenced panels, Figure 2B, and S2B, where non-COVID-19 preprints appear to have been screened more rapidly than non-COVID-19 preprints. Particularly, there appear to be more non-COVID-19 preprints with a screening time of 0 or 1 days. As the distributions of screening times are not normally distributed (and cannot be close to 0 as there is no negative screening time), providing the median rather than mean - and doing so separately for bioRxiv and medRxiv - and providing a fitting non-parametric test (e.g.: ranksum) could more accurately describe the overall trends in the data.

The original manuscript contained an error in analysis regarding this result. This has been amended in the reanalysis, and we now report little difference in screening time between COVID-19 and non-COVID-19 preprints, instead finding a larger different between bioRxiv and medRxiv servers. Estimation of effects of both server and preprint type and their interaction requires a two-way ANOVA, for which there is no simple nonparametric version. We have now added a supplemental table containing nonparametric descriptive statistics (see Supplemental Table 1).

The analysis of the subsequent paragraph, between lines 142-146, the additional variability in team size, a half sentence which separates bioRxiv vs. medRxiv may help to clarify how much of the variability would stem from different team sizes in different academic fields (e.g.:, molecular biology vs. clinical research).

We have now added a supplemental table containing differential descriptive statistics stratified by preprint server and refer to this in this paragraph: “Though little difference was seen in number of authors between preprint servers (Supplemental Table 1)”

"Additionally, India had a higher representation among COVID-19 authors specifically using preprints for the first time compared to non-COVID-19 posting patterns." The visual impression from the figure is that the absolute numbers for India are very small, opening the possibility that the difference is not statistically significant. Adding a statistic test to the statement would prevent those thoughts.

Supporting statistical tests have now been conducted; we find significant association for India as well as other countries. Full details of these tests are contained within Supplemental Table 4.

Within Figure S2, panel D appears on the left-hand side from panel C, which is opposite to the reading direction (left to right) common in most scientific publications.

We have replaced all figures in the manuscript and have confirmed there are no formatting issues.

The discussion section claims a cultural shift regarding media. However, it remains unclear whether there was a long-lasting effect, as implied through "shift", which remained after April 2020. Further it remains unclear whether there was a conceptual change in the way media would operate that has been enabled by preprints, or whether also in the early days of other epidemics media used essentially any source outside of scientific journals to obtain information that they would include in their coverage (e.g.: interviews with scientists, reports of local health agencies….).

Our updated dataset now includes data beyond April 2020 and confirms that there has been a long-lasting change in how the media are utilising preprints. Media coverage of COVID-19 preprints has remained consistent since April 2020, experiencing a slight increase again in October 2020. Moreover, this has now been investigated by other studies [7]. We do not address the media coverage in other epidemics as that would be beyond the scope of this current work.

The statement on politicization on science needing to be "prevented at all costs" could be a little bit more specific to avoid reading it in a possibly unintended manner. For instance, one could argue that arguments among politicians should be based on science, that scientists should focus their research on problems identified by societies and their representatives, and that academics should also care about gender or racial injustice that is present in their societies to avoid that these are manifested through educational systems.

We have revised our wording to be more specific: “Moreover, the politicisation of public health research has become a polarising issue and more must be done to combat this; scientific advice should be objective and supported by robust evidence. Media outlets and politicians should not use falsehoods or poor-quality science to further a personal agenda”

Very, very minor point of curiosity - please fully ignore for any practical concerns unless already considered somehow by authors: The manuscript very understandably focuses on academic science. Would the findings also extend to non-academic preprint servers that scientists should ignore for many good reasons, such as viXra.org?

This is not something we have looked into but is a very interesting point. Of note, one of the limitations in our study was the ease of data availability. Only bioRxiv and medRxiv had easily accessible data and we relied on the generous supply of data from the other preprint servers. As such it would be a more intensive effort to perform similar analyses on non-academic preprint severs.

## Reviewer #4:

This paper focuses on summarizing various attributes, bibliometrics, and altmetrics of preprints pertaining to the COVID-19 pandemic. Overall, I think the manuscript is thoughtful and thorough, and provides a timely overview of how the pandemic has impacted scientific publishing and vice versa. Even beyond the pandemic, this should prove to be a useful point of reference in the ongoing debate surrounding preprints, open science, and peer review.

While the descriptive statistics and univariate tests provide a nice backdrop for thinking about the unprecedented changes to publishing practices induced by COVID-19, I'd like to see the authors attempt to address some more challenging hypotheses and apply some slightly more sophisticated multivariate statistical analyses to support their conclusions.

MAJOR COMMENTS

The authors allude to "anecdotal observations that preprints are being used to share more works-in-progress" (line 192) as the reason COVID-19 preprints tend to be shorter in length--is this based on the authors' own anecdotal evidence, or are there references that can be cited here? "Works-in-progress" implies the research published was not as rigorous as in non-COVID-19 research--although there are certainly examples where results in a preprint were "half-baked", this scenario should be differentiated from studies in which authors are simply sharing results incrementally in short reports rather than waiting to accumulate multiple results before sharing. This practice is something various publishers have tried to promote over the years (e.g., Cell Reports [https://www.cell.com/cell-reports/aims)--perhaps](https://eur01.safelinks.protection.outlook.com/?url=https%3A%2F%2Fwww.cell.com%2Fcell-reports%2Faims)--perhaps&data=04%7C01%7C%7C02799805ed204eab3d5608d88a2cf9f9%7C569df091b01340e386eebd9cb9e25814%7C0%7C0%7C637411273089726268%7CUnknown%7CTWFpbGZsb3d8eyJWIjoiMC4wLjAwMDAiLCJQIjoiV2luMzIiLCJBTiI6Ik1haWwiLCJXVCI6Mn0%3D%7C2000&sdata=SqCC24vKzmzG2uRDkBgHc20bFJc8SqXFQvfukr5rP0o%3D&reserved=0) the authors could tease out the "work in progress" versus "short report" hypotheses by testing if the word count of COVID-19 preprints is associated with higher rates of

publication or faster turnaround in peer-reviewed journals.

As in our response to Reviewer 1 and 2, we have now used multivariate regression to investigate variables associated with word count, including publishing delays. We find that publishing delay is positively associated with word count for both COVID-19 and non-COVID-19 preprints, with a stronger relationship for COVID-19 preprints (Supplemental Model, Supplemental Table 3). We have removed the sentence “anecdotal observations that preprints are being used to share more works-in-progress". We have since performed additional work (see Rebuttal Fig 2) which, suggests that these shorter preprints are largely complete works or independent pieces of data; i.e. short reports rather than works-in-progress.

I can think of a few other explanations for the shorter length of COVID-19 preprints that could be tested, e.g., perhaps epidemiology papers tend to be shorter than papers in other fields of study and epidemiological studies are overrepresented among the COVID-19 preprints. Similarly, the authors mention elsewhere that relatively more COVID-19 preprints tend to have only a single author, which could also partially explain the shorter length, since there are ostensibly fewer person-hours invested in the writing than a multi-author study. There might also be cultural differences that contribute to paper length--do authors from China or India (the two countries noted as having the greatest increase in representation among COVID-19 preprints) tend to write shorter papers overall? Later, the authors recognize that COVID-19 preprints contain fewer references, which could itself contribute to the shorter length, as there is less need to situate new results against existing

literature (in which case we might expect these preprints to have gotten longer as the pandemic has progressed). It should be straightforward to apply a regression model to assess the relationship between paper length and COVID-19 focus, adjusting for topic, author count, author country, date posted, etc.

Our supplementary multivariate regression addresses all these factors, and finds that although bioRxiv subject categorisation, authorship team size and country of corresponding author have detectable impacts on preprint lengths, a substantial and significant difference remains in word counts of COVID-19 preprints versus non-COVID-19 preprints (Supplemental Model, Supplemental Table 2). As in our response to Reviewer 1 and 2, we have also examined references:word count ratios and find that references counts are in line with expectation based on word counts.

In the section "Extensive access of preprint servers for COVID-19 research", how much are the average number of abstract views/downloads influenced by outliers like the Santa Clara seroprevalence study and the withdrawn "uncanny inserts" study? More generally, are abstract views/downloads strongly correlated with attention on social media?

We believe that our boxplots show the impact of outliers – indeed, the Santa Clara seroprevalence study is an outlier in almost all aspects that we investigated. However, in our updated data, the impact of these early outliers has been dampened as the focus has shifted to more robust preprints. For example, the preprint suggesting genetic similar of SARS-CoV-2 inserts to HIV proteins has dropped from the second most-tweeted article to the fourth most-tweeted in our recent data. The impact on our reported rate ratios (abstract views = 18.2, downloads = 27.1) is minimal following exclusion of these two studies (abstract views = 17.7, downloads = 24.7), and moderate following exclusion of the top ten studies in each respective metric, although COVID-19 preprints remained highly elevated (abstract views = 14.2, downloads = 19.5). Statistical significance was unchanged in each case.

Lines 267-270: it would be good to provide some numbers here on the total number of original tweets and hashtags analyzed. Also, take some space to elaborate on why hydroxychloroquine is a controversial topic and why certain conspiracy theories have latched onto these top 10 most tweeted preprints. The wordcloud in Supplemental Fig 4A shows some extraordinary evidence of politicization that isn't mentioned, including the QAnon conspiracy theory ("qanon" and "wwg1wga"), xenophobia ("chinazi"), and US-specific right-wing populism ("maga", "foxnews", "firefauci") (I also think this figure could be moved to the main text). Given that the authors don't shy away from denouncing the politicisation of science as "a polarising issue [that] must be prevented at all costs" (line 409), this section feels much too short in its current form.

We have analysed the top 100 tweeted COVID-19 preprints and their associated hashtags (supplemental Fig 4). We have also expanded our discussion around the politicisation of COVID preprints and conspiracy theories.

The paper makes several references to research that is "poor-quality" or "controversial" but does not rigorously define or classify such preprints. With all of the data at hand, a cool deliverable might be to isolate particular attributes associated with low quality or propensity for controversy. Even some simple descriptive of a curated subset of such preprints would be interesting.

We agree that this would be a very interesting avenue to pursue. However, it is beyond the scope of the current study as we feel this would require a substantial investment of time to thoroughly address. Of note, we are about to submit a second study investigating differences between the preprinted and published versions of articles which begins to address this point (see Rebuttal Figure 2, response to reviewer 2).

Moreover, as an automated method of achieving this aim, we did attempt to use sentiment analysis of comments and tweets associated with each preprint. We used an adjusted Semantic Orientation CALculator (SO-CAL) lexicon, excluding scientific or infectious disease related terms (e.g., “respiratory”, “cellular”, “abstract”) and adjusting for valence shifters (i.e., context-altering words or phrases such as “not” or “anything but”). However, we found sentiment polarity to be a poor indicator of quality, as scores from individual tweets or comments showed high variability within-preprint, leading to near-neutral mean polarity scores per preprint even for the top ten tweeted preprints (Rebuttal Figure 4), several of which were clearly negatively received by scientific or public audiences (Table 2) . Additionally, external validation using citation contexts for preprints using date from scite (<https://scite.ai/>) showed little relationship between sentiment polarity and citations supporting or contracting (e.g., Rebuttal Figure 5)

**Rebuttal Figure 4.** Distribution of sentiment polarity scores associated with the top 10 tweeted COVID-19 preprints. Each individual data point represents a single tweet, with polarity score calculated as the mean polarity score across all period-separated sentences, using the SO-CAL lexicon for scoring.

**Rebuttal Figure 5.** Scatterplot of proportion of citations to preprints labelled as “contradicting” by scite and grand mean sentiment polarity score of tweets per preprint (calculated by averaging polarity score across all sentences within-tweet, before averaging the resulting averages within-preprint).

I understand the following request might not be feasible, so consider it optional, but it would be great to see the results of this paper updated to include preprints published more recently than April 30--there's a full 6 months of data that are ignored (spanning the first big peak of cases in the US in July and the ongoing second wave), and there are potentially some really interesting stories to tell--not just about the overall characteristics of COVID-19 preprints, but how they have evolved over time.

We updated the dataset to include all preprints deposited to bioRxiv and medRxiv from 1st January 2020 to 31st October 2020 (see also responses to other reviewers). We have also expanded our discussion to include comments on how the characteristics and usage of COVID preprints have changed over time.

MINOR COMMENTS

Fig 1a: since case and death counts are shown together on the same panel, this figure would be more readable with the y-axis on a log scale

Y-axis of Fig 1A is now on a log scale

Fig 1b: Since all of the other figures use the same color scheme for COVID-19 preprints vs non-COVID preprints, it would be better to use a different color scheme to describe preprints vs. journal articles here.

Color scheme of Figure 1B has been updated

Line 55: closing parenthesis should come after "case"

We have corrected this in text.

Line 70: "certified" has strong connotations--better to just say preprints have not gone through formal peer review yet

We have removed this from the manuscript

Line 98: Maybe say "*at least* 186 articles," unless the authors are certain this is an exhaustive count

We have altered our wording throughout the manuscript

Line 109: Were any preprints that went on to be published in peer-reviewed journals double-counted among the 16,000 COVID-19 articles mentioned here?

Yes, if an article appears as both a preprint and published in a journal then it was counted as both.

Line 121: spell out OASPA acronym

We have corrected this in text.

**References**

1.	Sevryugina YV, Dicks AJ. Publication practices during the COVID-19 pandemic: Biomedical preprints and peer-reviewed literature. bioRxiv. 2021; 2021.01.21.427563. doi:10.1101/2021.01.21.427563

2.	Horbach SPJM. Pandemic Publishing: Medical journals drastically speed up their publication process for Covid-19. bioRxiv. 2020; 2020.04.18.045963. doi:10.1101/2020.04.18.045963

3.	Fang Z, Costas R. Studying the accumulation velocity of altmetric data tracked by Altmetric.com. Scientometrics. 2020;123: 1077–1101. doi:10.1007/s11192-020-03405-9

4.	Haustein S, Bowman TD, Costas R. When is an article actually published? An analysis of online availability, publication, and indexation dates. ArXiv150500796 Cs. 2015 [cited 22 Jan 2021]. Available: http://arxiv.org/abs/1505.00796

5.	Eisen MB, Akhmanova A, Behrens TE, Weigel D. Publishing in the time of COVID-19. eLife. 2020;9: e57162. doi:10.7554/eLife.57162

6.	Carneiro CFD, Queiroz VGS, Moulin TC, Carvalho CAM, Haas CB, Rayêe D, et al. Comparing quality of reporting between preprints and peer-reviewed articles in the biomedical literature. bioRxiv. 2020; 581892. doi:10.1101/581892

7.	Fleerackers A, Riedlinger M, Moorhead L, Ahmed R, Alperin JP. Communicating Scientific Uncertainty in an Age of COVID-19: An Investigation into the Use of Preprints by Digital Media Outlets. Health Commun. 2021;0: 1–13. doi:10.1080/10410236.2020.1864892

8.	Abdill RJ, Blekhman R. Tracking the popularity and outcomes of all bioRxiv preprints. Pewsey E, Rodgers P, Greene CS, editors. eLife. 2019;8: e45133. doi:10.7554/eLife.45133

9.	Fraser N, Momeni F, Mayr P, Peters I. The relationship between bioRxiv preprints, citations and altmetrics. Quant Sci Stud. 2020; 1–21. doi:10.1162/qss_a_00043

10.	eLife. New from eLife: Invitation to submit to Preprint Review. In: eLife [Internet]. eLife Sciences Publications Limited; 13 May 2020 [cited 13 May 2020]. Available: https://elifesciences.org/inside-elife/d0c5d114/new-from-elife-invitation-to-submit-to-preprint-review

11.	Lloyd-Smith JO. Maximum Likelihood Estimation of the Negative Binomial Dispersion Parameter for Highly Overdispersed Data, with Applications to Infectious Diseases. PLOS ONE. 2007;2: e180. doi:10.1371/journal.pone.0000180

12.	ASAPbio. Preprint authors optimistic about benefits: preliminary results from the #bioPreprints2020 survey. In: ASAPbio [Internet]. 27 Jul 2020 [cited 1 Feb 2021]. Available: https://asapbio.org/biopreprints2020-survey-initial-results
