# Supplementary material for: The evolving role of preprints in the dissemination of COVID-19 research and their impact on the science communication landscape
Source: PLoS Biol. 2021 Apr 2;19(4):e3000959. doi: 10.1371/journal.pbio.3000959 (PMC8046348; doi:10.1371/journal.pbio.3000959)
Supplement: Attachment — Submitted filename: Reviewer comments_Resubmission.docx [file pbio.3000959.s011.docx]

Dear Dr Coates,

Thank you for submitting your revised Research Article entitled "Preprinting the COVID-19 pandemic" for publication in PLOS Biology. I have now obtained advice from three of the original reviewers and have discussed their comments with the Academic Editor. 

Based on the reviews, we will probably accept this manuscript for publication, provided you satisfactorily address the remaining points raised by the reviewers. Please also make sure to address the following data and other policy-related requests.

IMPORTANT:

a) Please attend to the remaining requests from reviewers #1 and #3.

b) Please could you choose a more informative Title. We suggest something like "Analysing the role of preprints in the dissemination of COVID-19 research and their impact on the science communication landscape," but please see the relevant comment by reviewer #1 and feel free to choose something that you think reflects the analysis and findings.

c) Please supply a blurb in the box in the submission form.

d) Many thanks for providing the data and code so fully in Github and Zenodo. Please could you cite the URLs/DOIs clearly in all relevant main and supplementary Figure legends (e.g. "The data underlying this Figure may be found in https://github.com/preprinting-a-pandemic/pandemic_preprints and https://zenodo.org/record/4501924").

As you address these items, please take this last chance to review your reference list to ensure that it is complete and correct. If you have cited papers that have been retracted, please include the rationale for doing so in the manuscript text, or remove these references and replace them with relevant current references. Any changes to the reference list should be mentioned in the cover letter that accompanies your revised manuscript.

We expect to receive your revised manuscript within two weeks.

To submit your revision, please go to https://www.editorialmanager.com/pbiology/ and log in as an Author. Click the link labelled 'Submissions Needing Revision' to find your submission record. Your revised submission must include the following:
-  a cover letter that should detail your responses to any editorial requests, if applicable, and whether changes have been made to the reference list
-  a Response to Reviewers file that provides a detailed response to the reviewers' comments (if applicable)
-  a track-changes file indicating any changes that you have made to the manuscript. 

NOTE: If Supporting Information files are included with your article, note that these are not copyedited and will be published as they are submitted. Please ensure that these files are legible and of high quality (at least 300 dpi) in an easily accessible file format. For this reason, please be aware that any references listed in an SI file will not be indexed. For more information, see our Supporting Information guidelines:
https://journals.plos.org/plosbiology/s/supporting-information  

*Published Peer Review History*
Please note that you may have the opportunity to make the peer review history publicly available. The record will include editor decision letters (with reviews) and your responses to reviewer comments. If eligible, we will contact you to opt in or out. Please see here for more details:
https://blogs.plos.org/plos/2019/05/plos-journals-now-open-for-published-peer-review/

*Early Version*
Please note that an uncorrected proof of your manuscript will be published online ahead of the final version, unless you opted out when submitting your manuscript. If, for any reason, you do not want an earlier version of your manuscript published online, uncheck the box. Should you, your institution's press office or the journal office choose to press release your paper, you will automatically be opted out of early publication. We ask that you notify us as soon as possible if you or your institution is planning to press release the article.

*Protocols deposition*
To enhance the reproducibility of your results, we recommend that if applicable you deposit your laboratory protocols in protocols.io, where a protocol can be assigned its own identifier (DOI) such that it can be cited independently in the future. For instructions see: https://journals.plos.org/plosbiology/s/submission-guidelines#loc-materials-and-methods 

Please do not hesitate to contact me should you have any questions.

Sincerely,

Roli Roberts


Roland G Roberts, PhD,
Senior Editor,
rroberts@plos.org,
PLOS Biology


------------------------------------------------------------------------
DATA NOT SHOWN?

- Please note that per journal policy, we do not allow the mention of "data not sown", "personal communication", "manuscript in preparation" or other references to data that is not publicly available or contained within this manuscript. Please either remove mention of these data or add figures presenting the results and the data underlying the figure(s).

------------------------------------------------------------------------

REVIEWERS' COMMENTS:

**Dear Editor,**

**Thank you for the additional opportunity to revise our manuscript. We have responded to the reviewers below. We would like to thank all reviewers for their thoughtful and helpful comments.**

Reviewer #1:

This revision by Fraser et al. is an interesting analysis and a valuable contribution to the field. They have addressed all of my major concerns, and the expansion of the dataset through October has enabled them to provide a much more comprehensive characterization of the relevant patterns. There are a few minor issues left:

MINOR NOTES:

I defer to the editor on whether this is a concern, but the current title, "Preprinting the pandemic," does not seem as descriptive as it could be. It reads as a feature headline but doesn't reveal any of the findings, nor does it describe the analysis in a useful way.

**We have now changed the title to be more informative: “The evolving role of preprints in the dissemination of COVID-19 research and their impact on the science communication landscape”**

Line 218: The paper states, "Critics have previously raised concerns that by forgoing the traditional peer-review process, preprint servers could be flooded by poor-quality research." A citation would be helpful here.

**We have added two supporting references here (now line 223); reference 20, Bagdasarian N, Cross GB, Fisher D. Rapid publications risk the integrity of science in the era of COVID-19. BMC Med. 2020;18: 192. doi:10.1186/s12916-020-01650-6 & reference 21, Sheldon T. Preprints could promote confusion and distortion. Nature. 2018;559: 445–446. These highlight both concerns with preprints and concerns with a rushed peer-review process.**

Lines 245-253: A lot of space and effort is spent here explaining that downloads for individual preprints taper off over time—this would be a valuable visualization to add, perhaps as a panel in Figure 5. It would help evaluate whether the average "debut month" was shrinking over time, which could indicate popular interest in COVID is waning. An example of a "downloads in first month" figure is available as Figure 2, figure supplement 3(a) in Abdill & Blekhman 2019 [1]—the x-axis could be something like "Month posted," and the y-axis would be "Downloads in first month." Using a visualization such as a box-and-whisker plot could illustrate how many downloads were received in, say, March, for preprints posted in March, followed by downloads in April for preprints posted in April, and so on.

**We have added two panels to Supplementary Figure 3 (new panels A and B) which show downloads in the calendar month in which the preprint was posted. The new figures show that the difference between the groups (COVID-19 versus non-COVID-19) reduced over time: COVID-19 preprints posted in the early months of the pandemic received disproportionately more views/downloads compared to non-COVID-19 preprints, than those posted later. Lines 246-257 have also been edited to reflect this. A major caveat is that usage data from bioRxiv and medRxiv are limited to calendar months, meaning that preprints posted later in the month will have less time to accrue views than those earlier in the month. Unfortunately we do not have finer-grained (e.g. daily) data available at this time.**

Figure 1: In panel C, it's difficult to see differences between the smaller servers, particularly because the number of segments means the colors are all very similar. It might be better to push the 7 smallest ones into the "other" bucket. This is not a critical issue and we leave it to the authors and editors whether to make this change.

**We decided to leave the smallest categories in the legend, as the names (e.g. “PsyArXiv”, “ChemRxiv”, “SocArXiv”) also provide useful information to the reader on the different disciplines where preprints have been published.**

Figure 3: In panel B, it's difficult to compare the two categories because of the dramatically different counts. The distributions (as in panel A) would be much more informative, particularly because the difference in distributions seems to be the most relevant result.

**We updated panel B of Figure 3 to show proportions instead of absolute numbers of preprints posted per country.**

Figure 6: The content of Panel E is limited by the poor match between the data and the scale of the y-axis. While it's logical that all panels would have the same y-axis, the primary comparison for readers doesn't seem to be between panels, but within them. The authors might consider altering the y-axis of this panel to make the differences easier to see.

**We changed the scale of the y-axis of Panel E to allow easier comparison between the data series.**

Several panels in figures 2, 4, 5 and 6 appear to use German abbreviations for month names, while others use English abbreviations. I'm not aware of specific language requirements from the journal, but consistency would be helpful.

**This was due to one of the authors default R settings – abbreviations are now in English.**

References:
[1] Abdill & Blekhman 2019. https://doi.org/10.7554/eLife.45133


Reviewer #3:

Fraser et al. present a series of good improvements for an already interesting manuscript.

I hope that prior publication, they could remove the last remnant of my prior main criticism - namely the reference to a "paradigm shift" contained in the abstract. The phrase "cultural shift" which they now use in the discussion section is very appropriate and fitting as their findings document an (important) shift in publication practices. In contrast, the original meaning of "paradigm shift" within studies of science refers to the way scientists probe phenomena. Kuhn emphasized this point in the foreword of later editions of The Structure of Scientific Revolutions as he noted that his phrase of "paradigm shift" had already become read in a more general and unintended manner by some. Similarly - at least for genes - research on COVID-19 appears to often use research patterns of the past (Stoeger et Amaral, eLife 2020). This would argue against a "paradigm shift" in its original sense.

**We have now changed this in the abstract from “paradigm shift” to “cultural shift”. We thank the reviewer for their comments.**

Other:

Figure labels for months use German abbreviations.

**This was due to one of the authors default R settings – abbreviations are now in English.**

While the authors have now greatly clarified points about the disproportional usage of preprint servers for COVID-19 this argument could possibly be extended in a half sentence that extends beyond their share in preprint servers, relative to other pandemics, toward the comparison of the share of COVID-19 in preprint servers against their share in journals. Such an option would seem supported by an approximate estimate of there being 100,000 COVID-19 publications according to LitCovid, and there being around 2.5 million papers added to MEDLINE every year (thus the ~25% of COVID-19 publications in preprint servers would exceed the ~4% anticipated for journals).

**We added some text to address this point (lines 98-100). As the numbers of preprints we report in this section is based on a variety of multidisciplinary preprint servers (i.e. not limited to biomedical fields), we also reported numbers of journal articles without narrowing down by discipline – we therefore used data from Dimensions, consistent with other parts of the paper, rather than MEDLINE.**

The reformulated text around lines 251 now seems to yield the originally intended message of the authors, which I had not noted in the initial review. As the analysis of this paragraph now stands, I would see their current suggestions as only one of two different options. The other option would be that COVID-19, but not non-COVID-19, preprints are subjected to an additional time-dependent factor (which one could suspect to be the changes of the number of publications available in the literature through journals). I believe that this point could be clarified by formulating the sentence again in a more general manner, or by doing an additional analysis (e.g. as the authors will have at least two time-stamped data queries, with one corresponding to the original submission, and the other corresponding to the update of the revision).

**This comment is addressed in the response to a similar point of Reviewer 1 (Lines 245-253).** **The same trends in abstract view and PDF download data is observed when limiting view and usage data to the month in which the preprint was posted (see new panels A and B in Supplementary Figure 3).**


Reviewer #4:

The authors have thoroughly addressed all of my previous comments.
